# Multimodal pain therapy for persistent idiopathic facial pain - a pilot study

**DOI:** 10.1186/s13030-022-00254-1

**Published:** 2022-12-09

**Authors:** Zita Foerster, Barbara Kleinmann, Nadine Schlueter, Kirstin Vach, Tilman Wolter

**Affiliations:** 1 Interdisciplinary Pain Center, Medical Center-University of Freiburg, Faculty of Medicine, University of Freiburg, Breisacher Str, 10779106 Freiburg, Germany; 2 Division for Cariology, Department for operative Dentistry and Periodontology, Center for Dental Medicine, Medical Center-University of Freiburg, Faculty of Medicine, University of Freiburg, Breisacher Str, 10779106 Freiburg, Germany; 3 Department of Operative Dentistry, Periodontology and Preventive Dentistry, Hannover Medical School, Hannover, Germany; 4 Institute of Medical Biometry and Statistics, Medical Center-University of Freiburg, Faculty of Medicine, University of Freiburg, Breisacher Str, 10779106 Freiburg, Germany

**Keywords:** Persistent idiopathic facial pain, Chronic pain, Multidisciplinary pain treatment

## Abstract

**Objective:**

Persistent Idiopathic Facial Pain (PIFP) is a pain syndrome with missing evidence-based therapy recommendations. According to the biopsychosocial pain model, multidisciplinary pain treatment (MPT) offers a promising therapeutic option for chronic pain syndromes. MPT is an interprofessional treatment procedure, consisting of medical, physiotherapeutic and psychotherapeutic treatment units, which has not yet been studied in PIFP.

**Methods:**

This retrospective study included 25 patients with PIFP, who had been treated with MPT. Pain intensity on the numerical rating scale (NRS), perceived disability, habitual well-being, as well as anxiety/depression and stress scales were recorded. Moreover, the patients evaluated the efficacy of each type of the single therapeutic interventions.

**Results:**

There was a highly significant decrease in the characteristic pain intensity. Also habitual well-being improved significantly, as did anxiety and depression. The perceived disability and stress also improved, but without statistical significance. Physiotherapy was rated as the most effective therapeutic unit. Among the medical measures, consultations took first place (40% of the participants). Nearly three-fourths of the patients (72%) would recommend MPT.

**Conclusion:**

The present study shows beneficial outcomes in patients with PIFP following MPT. Patients evaluate physiotherapeutic treatment as particularly efficacious. Therefore, MPT can be considered as a therapeutic option in patients with PIFP.

## Introduction

Persistent Idiopathic Facial Pain (PIFP), formerly called atypical facial pain, was described for the first time in 1924 [1] and remains a controversial and poorly-understood pain syndrome. Many physicians and dentists consider PIFP the last resort in making the diagnosis or even as a” wastebasket diagnosis “[2, 3]. Moreover, this pain syndrome is quite uncommon with an annual incidence of 4.4 per 100,000 [4] and a lifetime prevalence of 0.03 [5].However, it is obvious that there are patients with chronic facial pain, whatever the diagnosis might be labelled [3].

The International Headache Society (IHS) describes PIFP as “Persistent facial pain that does not have the characteristics of [...] cranial neuralgias [...] and is not attributed to another disorder” [6]. Moreover, four diagnostic criteria have to be fulfilled (Table 1). Compared to the definitions of other pain societies, there are certain discrepancies, for instance the presence of concomitant symptoms such as dysesthesia [7]. Furthermore, individual pain societies did not accept the diagnosis of PIFP [8]. Therefore, a revision and standardization of the description of the facial pain symptoms was demanded [9, 10]. Meanwhile an international classification of orofacial pain has been published [11], which however was not yet available at the time of initiation of the present study and the present study therefore still uses the International Classification of Headache Disorders-II (ICHD-II) criteria.

**Table 1:** Diagnostic criteria of PIFP according to IHS Classification 2nd edition [5]

A. Pain in the face, present daily and persisting for all or most of the day, fulfilling criteria B and C	
B. Pain is confined at onset to a limited area on one side of the face^a^, and is deep and poorly localized	
C. Pain is not associated with sensory loss or other physical signs	
D. Investigations including X-ray of face and jaws do not demonstrate any relevant abnormality	

^a^Pain at onset is commonly in the nasolabial fold or side of the chin, and may spread to the upper or lower jaw or a wider area of the face and neck.

To date, there are no evidence-based recommendations for the pharmacological treatment of PIFP [7]. Nonetheless, tricyclic antidepressants such as amitriptyline are considered as first-choice medications [12]. In addition, it is emphasized that surgical procedures should only be based on a specific diagnosis [13]. Moreover interventional procedures such as sphenopalatine ganglion injections and pulsed radiofrequency [14], botulinum toxine injections [15], radiofrequency of the ganglion gasseri [16] or recently peripheral nerve field stimulation [17] have been described.

PIFP is a chronic pain syndrome. Chronic pain has to be regarded as an entity of its own, which cannot be characterized solely by the duration of its presence. Affective, sensory, psychological and social factors which can cause or sustain the pain syndrome must also be considered [18]. All these interactions are integrated in the biopsychosocial pain model [19], which is the basis of the current understanding of chronic pain and therapeutic concepts derived therefrom.

Multidisciplinary pain treatment (MPT) is an effective procedure in the treatment of chronic pain [20]. According to the definition of the German Pain Society (Deutsche Schmerzgesellschaft, DSG), MPT comprises medical, psychological and physiotherapeutic measures enabling a team of therapists from different specialties to pursue a common therapy goal [21]. Here, the central objective is functional restoration [21]. The term:” functional restoration” in conjunction with PIFP might seem rather uncommon. However, if PIFP is seen as a severe chronic pain syndrome, several psychological, social and biomechanical functions can be impaired, ranging from impaired mood over more frequent sick leave to an impaired motility of the cervical spine and accompanying neck pain and tenderness.

The present study sought to examine whether MPT could be an effective treatment option for PIFP and whether an amelioration of pain, depression and anxiety as well as habitual well-being would be found. To that end, 25 patients diagnosed with PIFP who had undergone MPT in the Interdisciplinary Pain Centre of the University Hospital (City Name) were examined regarding the effects on subjective perception of their pain state. Moreover an evaluation of the single therapeutic units was performed as a Patient Reported Outcome Measurement (PROM). Primary outcomes under study were pain, depression, anxiety and habitual well-being. Secondary outcomes were the individual ratings of each type of the single therapeutic interventions.

## Methods

### Patient selection

The present retrospective study was performed at the Interdisciplinary Pain Center, University Hospital (city name). The study was approved by the Ethics Committee of the University Hospital (city name) (No.473/16). The study was performed in accordance with the Declaration of Helsinki.

First, a preliminary selection of the potential study collective by means of electronic search of all discharge notes in the years 2009 to 2015 was performed, regarding the following ICD codes: R51, G50.0, G50.1, G50.9, G50.8, K10.8, K08.88. This first step served as a filter to preselect patients with orofacial pain syndromes. Then, the hits were individually controlled for the diagnosis PIFP. All clinical features of PIFP had to be present for inclusion in the study. Patient with competing diagnoses such as trigeminal neuralgia were excluded. According to common clinical terminology, terms such as “continuing” or “ongoing” were included, as were paraphrases of idiopathic such as “without identifiable cause” or “etiology not proven”. Also, the former diagnosis of atypical facial pain was included. Moreover, the clinical description in the files was checked regarding the presence of criteria of PIFP based on the IHS definition according to the ICHDII.

### Inclusion criteria

- diagnosed PIFP
- age between 18 and 80 years
- participation in a 5-week MPT between 01.01.2009 and 31.07.2016

### Exclusion criteria

- Facial pain not meeting criteria for PIFP
- Inability to perform telephone interview
- Incomplete questionnaire
- MPT of less than 5 weeks

### Multidisciplinary pain treatment

Multidisciplinary pain treatment was carried out according to the German Operations and Procedure Key (OPS) -Code 8–918 (interdisciplinary multidisciplinary pain treatment) over a five-week period in a day hospital setting. This program meets the requirements of the OPS and is therefore comparable to corresponding programs in Germany [22]. Treatment included medical, psychotherapeutic and physiotherapeutic measures. These were adapted individually for each patient and carried out by an interprofessional team. Treatment was discussed and harmonized during weekly team conferences. Medical treatment included counselling consultations and individual adjustment of the medication. Mostly tricyclic antidepressant and occasionally anticonvulsants were used. A preexisting medication was reduced more often than expanded. Individual and group physiotherapy was conducted. Within the group therapy the patients were instructed about procedures particularly movement exercises. Individual physiotherapy was performed depending on the patients’ particular situation and needs. Physiotherapy moreover included stretching exercises, trigger points treatment, friction massage manual therapy and Medical Training Therapy (MTT). MTT or exercise therapy is a form of physiotherapy. It is a targeted training, which includes joint training (mobilisation and coordination training), movement initiation, muscle training and prevention training to motivate health-promoting behavior.

**Fig. 1:**
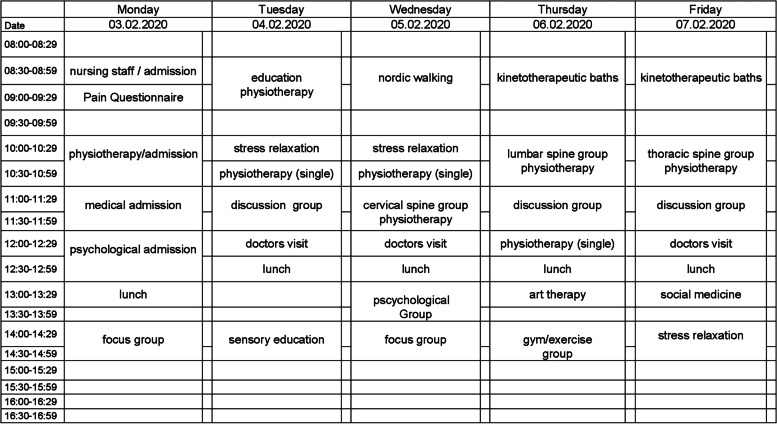
example of a weekly treatment schedule for MPT

Psychotherapy was also offered as both individual and group therapy. Psychotherapy was based on a behavioral approach. The identification of pain-sustaining cofactors, as well as acceptance of and coping with pain were topics of the therapy. Moreover, occupational therapeutic and sociomedical treatments were available as necessary. An example of a weekly schedule for individual patients is shown in fig. 1.

### Data acquisition

All data collected by telephone interview in the period between 05.12.2016 and 19.01.2017 and from the patient files] were included. Prior to the interview the patients gave informed consent to participation in the study. None of the patients stood under legal guardianship.

In the telephone interview, first the criteria of PIFP prior to and after MPT were asked. The telephone interview included pain scores (current, mean, maximal) on an 11-point (0–10) numeric rating scale (NRS) as principal primary outcome. Further depression, anxiety and stress were measured by the depression, anxiety and stress scale (DASS) [23]. This scale consists of 7 items each for depression, anxiety and stress. In each of these items 0–3 points can be reached. Total values above 10 indicate an increased probability of the presence of an anxiety or depressive disorder while values above 6 are suspicious for increased stress. A close correlation of the DASS scales to the BDI (Beck Depression Inventory) and the BAI (Beck Anxiety Inventory) has been shown by Nilges et al. (2015). The published Cronbachs α values were 0.91 for the depression scale and 0.78–0.8 for the anxiety and stress scales [24].

Perceived disability was measured with the disability score, a shortened version (3 items) of the 7-item Pain Disability Index (PDI) for the experience of impairment, in which scale items are rated on an 11-point scale ranging from 0 to 10. The mean value of these three items multiplied by 10 gives the value for the disability score.

The PDI was developed particularly for patients with pain [25–27]. Factorial validity, construct validity and sensitivity to change are empirically proven. Cronbach’s alpha was shown to be 0.90 [28, 29].. The German translation by Dillmann et al.(1994) consists of 7 items [30].

Habitual well-being was recorded after MPT by means of the MFHW (Marburger Fragebogen zum Habituellen Wohlbefinden), a 7-item questionnaire questionnaire on habitual health findings with a 6-point scale for each item [23, 24, 31]..

This questionnaire has proven a good internal consistency (Cronbach’s α = 0.91) and test reliability after an 8-week interval (rtt = 0.81). The one dimensional test scale is showed a good reproducibility and factor structure with an explanation of variance of at least 65%. The construct validity has been evidenced by correlations with indicators for chronification [32].

Further, patients were asked for their individual evaluation of the efficacy of the single therapeutic measures on an 11-point-scale (0 = not effective, 10 = very effective). The data acquisition by telephone interview ensured that there were no missing data. All interviews were conducted by the same person in order to minimize potential interviewer bias.

From the patient files, the discharge notes and the German pain questionnaire (Deutscher Schmerzfragebogen, DSF) [33], which patients routinely filled in prior to therapy, were examined. Data regarding pain intensity, depression, anxiety, stress, perceived disability and habitual well-being prior to therapy were also extracted from these questionnaires. Pre/post comparisons were only carried out with complete data. Data acquisition after therapy was carried out on average 3.5 ± 1.9 years after MPT.

### Power considerations

For our study, all patients matching to the study criteria were telephoned and 42 patients could be contacted. Finally, 25 patients could be included (Fig.1). With 25 patients, an α = 0.05 and a power of 0.8 an effect size of 0.58 can be detected for the characteristic pain rating as the principal outcome.

### Data analysis

First, data were controlled for normal distribution (Kolmogorov-Smirnov-Test). The analysis regarding age, sex, diagnosis, concomitant medication and therapies and prior operations was made by categorization and descriptive statistics (absolute frequencies (n) and percentages (%)). For the parameters, media were calculated prior to therapy and median values, minima and maxima, mean values and standard deviations as well as absolute and relative frequencies after therapy. The t-test for paired samples was used to examine the pre/post differences in case of normally-distributed values, the Wilcoxon rank sum test in case of missing a Gaussian distribution. Levels of significance were *p* < 0.05 (significant, *) and *p* < 0.01 (highly significant, **). A Bonferroni correction was calculated to correct multiple testing in case of measures with multiple single measures.

## Results

### Patients

**Fig. 2:**
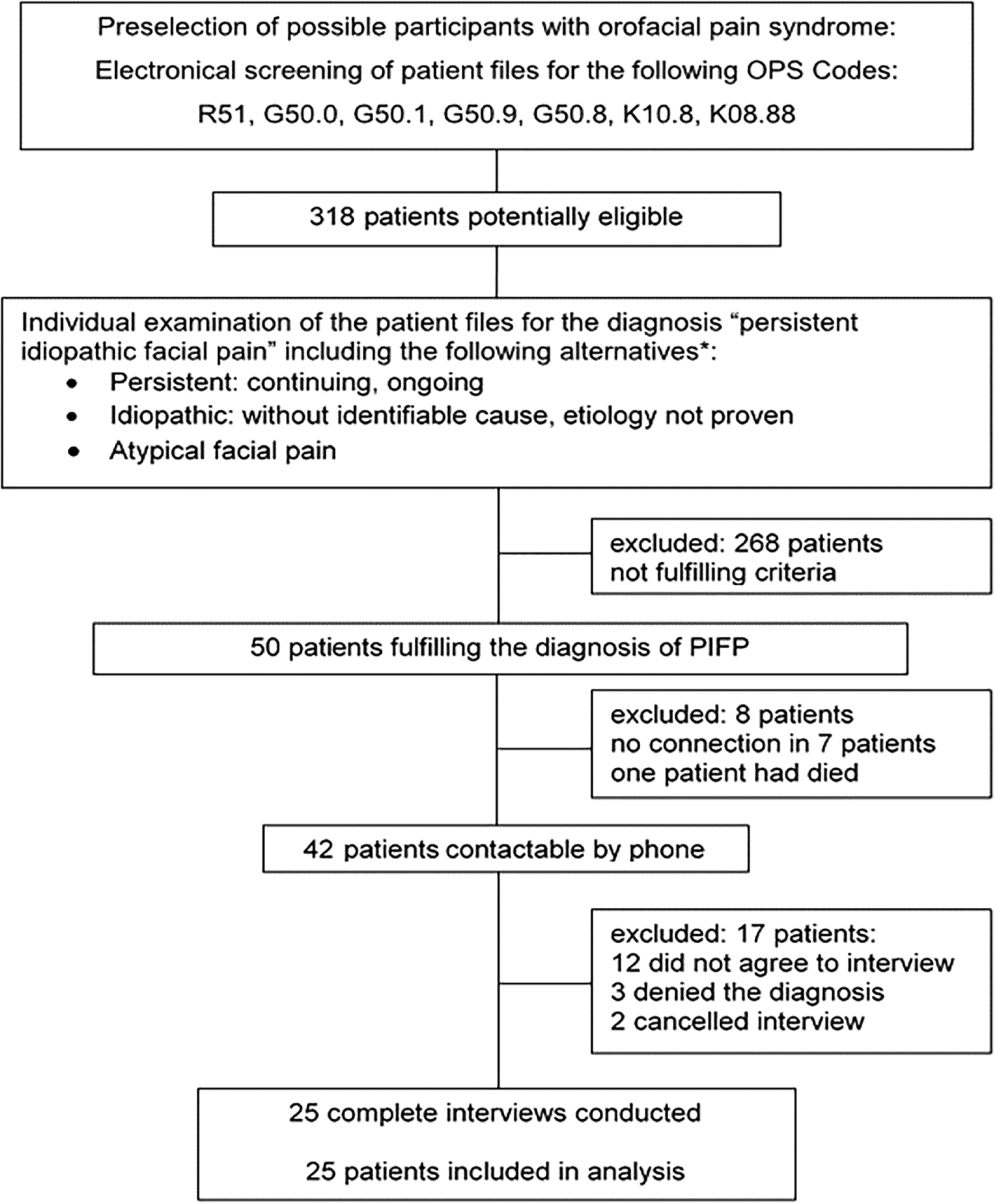
flow diagram of patients treated, patients eligible and patients analysed, *translation from German language

The study sample consisted of 25 patients (16 women/9 men). The mean age was 56.6 ± 12.7 years. The median duration of the pain anamnesis was 9.7 years, interquartile range (IQR) 5.7; 16.5 years (mean 11.6 years, minimum 1.6 years, maximum 32.0 years) (Table 2, fig. 2).

**Table 2:** Baseline characteristics of the patient collective: AD = antidepressants, AK = anticonvulsants, NL = neuroleptic agents, TQ = tranquilizers, MR = muscle relaxants, + = weakly effective, ++ = strongly effective, OMS = oral and maxillary surgery, TENS = ranscutaneous electric nerve stimulation, IQR = interquartile range

	patients (n)]	patients (%)]
Sex		
men	9	36%
women	16	64%
**Age [Years]**		
56.6 (SD12.67)		
**History duration [Years]**		
Median 9.6, (IQR 5.7,16.5 years)		
**ICD Coding**		
**G50.1** (atypical facial pain)	10	40%
**R51** (headache, facial pain)	10	40%
**G50.8** (disorders of the N. trigeminus)	1	4%
**G50.0** (trigeminal neuralgia)	1	4%
**K10.8** (mandibular disorders)	1	4%
**F45.41** (chronic pain disorder)	1	4%
**R51 + G50.0**	1	4%
**Pain medication**		
Opioids	8	36%
Strong	5	20%
Weak	3	12%
Non-opioid analgetics	13	52%
AD	13	52%
AK	12	48%
NL	1	4%
TQ	1	4%
MR	1	4%
**Concomitant disorders**		
internal	14	56%
degenerative	7	28%
neurologic	5	20%
pain syndrome	19	76%
psychiatric	24	96%
psychosocial	14	56%
physiotherapeutic	19	76%
concomitant to PIFP	7	28%
other	14	56%
**prior operations**		
head, face [excl. OMS]	10	40%
OMS	12	48%
other	20	80%
**adjunct therapies**		
specialist consultations	8	32%
TENS	6	24%
other	14	56%

### Outcome parameters

The characteristic pain intensity calculated from current, mean and maximal pain intensity decreased significantly after MPT (*p* = 0.001). Also all single pain measures decreased significantly after therapy (current pain: *p* = 0.038, mean pain: *p* = 0.026, highest pain, *p* = 0.046). The perceived impairment regarding daily living, leisure time and work, as measured by the disability score showed no statistically significant difference before and after therapy, neither in the three single areas, nor in the sum score. The sum score of the MFHW, which was calculated from seven single items showed a statistically significant improvement (p0 0.016) while the single items except the item “feeling comfortable” showed no statistically significant differences. The depression and anxiety, but not the stress scores showed a statistically significant decrease after MPT (depression: *p* = 0.02, anxiety: *p* = 0.005, stress: *p* = 0.70) (Table 3).

**Table 3:** Outcome characteristics: pain intensity on a 11-point scale, Characteristic pain = mean current, average and maximal on a 100-point scale, Habitual Well Being (Marburger Fragebogen zum habituellen Wohlbefinden, MFHW), Disability score, Depression, Anxiety and Stress measured with the HADS-D (Hospital Anxiety and Depression Scale) and the DASS (Depression-, Anxiety-Stress-Scale), * paired t-test, **Wilcoxon-Mann-Whitney-Test, ^a ^*p*-values using Bonferroni corrections, p-values of non-normally distributed data based on Wilcoxon-Mann-Whitney-Test, all effect sizes (Cohen’s d) and confidence intervals (95% CI) based on t-test for paired samples

Outcome Parameter	mean (SD)		median (IQR)		n	p	Cohen’s d	95% CI
	pre	post	pre	post				
Current pain			6.0 (4.0; 8.0)	5.0 (2.0; 6.0)	25	0.038**^a^		
Mean pain			6.0 (5.0; 8.0)	6.0 (3.5; 6.5)	24	0.026** ^a^		
Maximal pain			8.5 (7.625; 9.375)	8.0 (6.5; 9.0)	24	0.046** ^a^		
Characteristic pain intensity	69.1 (5.7)	55.1 (16.5)			23	0.001*	0.77	[0.34; 1.21]
MFHW (daily task)			2.0 (1.0; 3.75)	3.0 (2.0; 5.0)	21	0.99** ^a^		
MFHW (inwardly fulfilled)			1.0 (0; 2.75)	3.0 (1.0; 3.0)	20	0.34** ^a^		
MFHW (feeling comfortable)			1.0 (1.0; 5.0)	3.0 (2.0; 4.0)	20	0.030** ^a^		
MFHW (enjoying life)			1.0 (0; 2.0)	2.0 (1.0; 3.0)	20	0.11** ^a^		
MFHW (satisfied with work performance)			2.0 (0.25; 3.0)	2.0 (0; 3.5)	20	6.56** ^a^		
MFHW (satisfied with physical state)			0 (0; 1.75)	2.0 (0.5; 3.0)	20	0.073** ^a^		
MFHW (truelly happy)			2.0 (1.0; 3.0)	3.0 (1.5; 5.0)	20	0.27** ^a^		
MFHW total	10.8 (7.6)	17.6 (8.3)			20	0.016*	−0.59	[−1.06; −0.12]
Disability Score Daily living	3.7 (2.9)	4.7 (2.9)			20	0.59* ^a^	−0.27	[− 0.68; 0.15]
Disability Score Leisure time	7.0 (2.5)	5.4 (2.7)			20	0.074* ^a^	0.48	[0.07; 0.89]
Disability Score Work	6.3 (2.7)	5.6 (3.3)			20	0.897* ^a^	0.21	[−0.20; 0.63]
Disability Score total	56.9 (23.4)	52.3 (25.6)			20	0.42*	0.16	[−0.25; 0.58]
Depression	9.5(4.3)	7.1 (5.5)			25	0.02*	0.50	[0.08; 0.91]
Anxiety	7.8 (4.9)	4.9 (4.1)			25	0.005*	0.62	[0.21; 1.03]
Stress	9.9 (4.0)	8.7 (3.8)			7	0.70*	0.59	[−0.34; 1.51]

### Evaluation

**Fig. 3:**
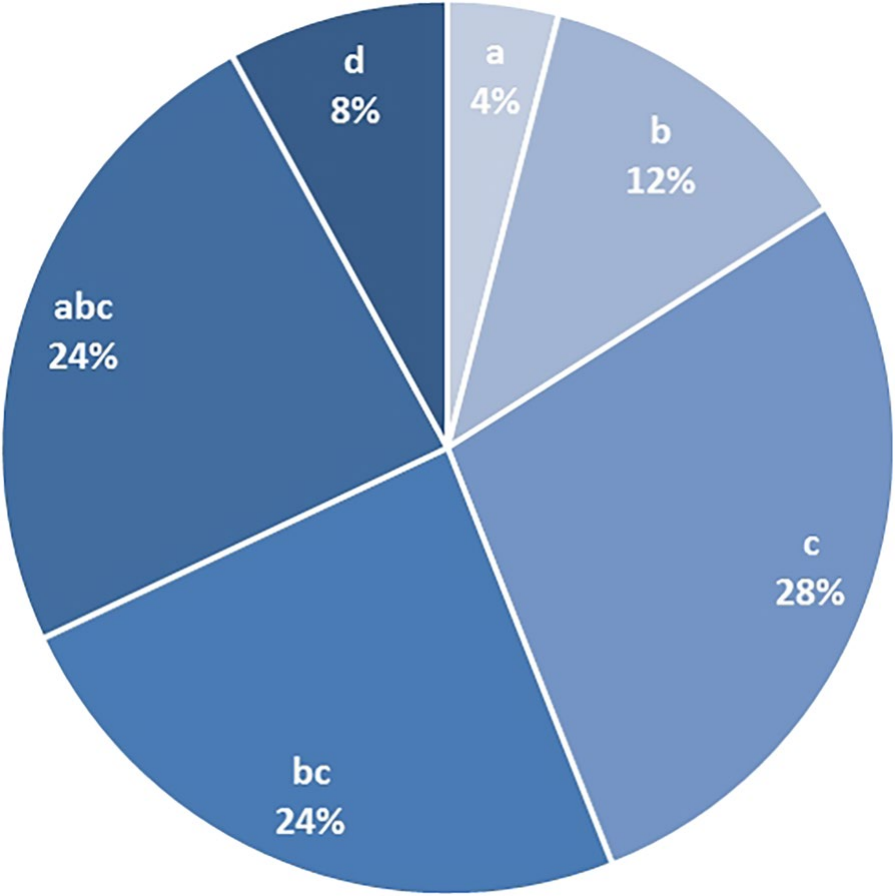
Evaluation of the most effective therapeutic measure, a = medical treatment, b = psychotherapy, c = physiotherapy, d = no measure

**Fig. 4:**
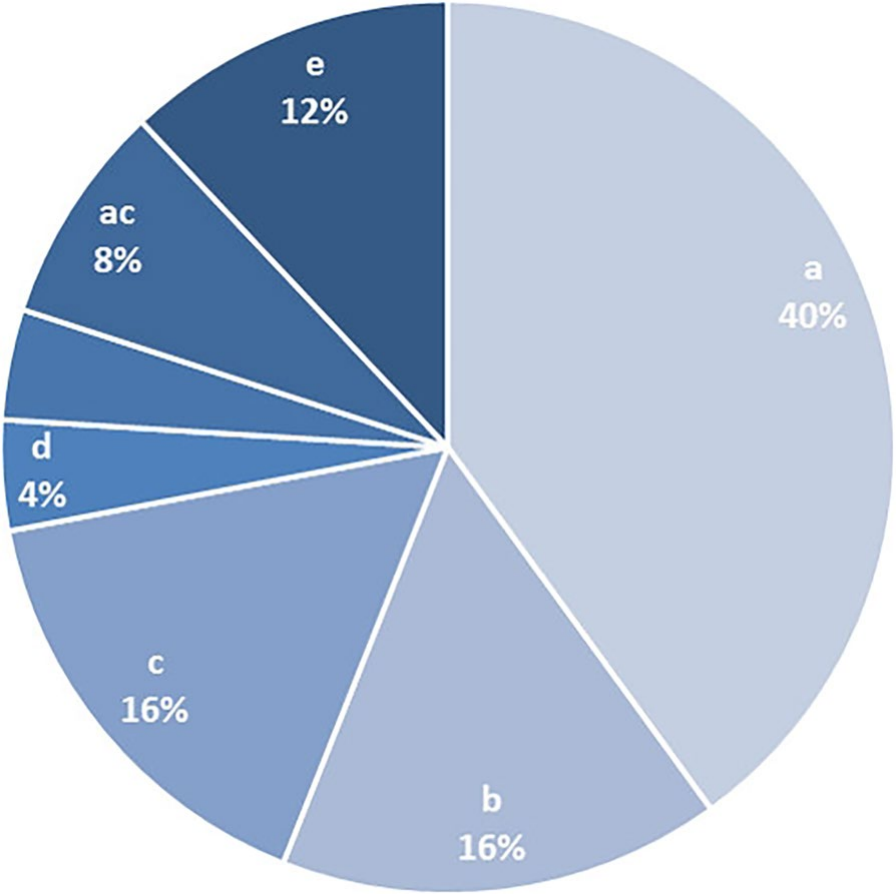
Evaluation of the most effective medical measure, a = consultations, b = education, c = medications, d = nerve infiltrations, e = no measure

The evaluation included the question of which was the most effective therapeutic measure of MPT. The distribution of answers was as follows: physiotherapy 7 (28%), a combination of physiotherapy and psychotherapy 6 (24%), a combination of psychotherapy, physiotherapy and medical treatment 6 (24%), psychotherapy alone 3 (12%), no single measure 2 (8%) and medical treatment alone 1 (4%) (fig. 3). The evaluation of the efficacy of the single measures on an 11-point scale for the single measures was as follows: medical treatment 5.5 ± 3.1, psychotherapy 6.2 ± 3.5 and physiotherapy 8.1 ± 2.1. Among the medical therapies, 10 (40%) of the patients rated medical consultations as effective, medications and education were rated as equally effective by 4 (16%) (fig. 4). Three (12%) of the patients rated no therapy as effective and 2 (8%) chose the combination of medical therapy and medical consultations. One (4%) each rated nerve infiltrations or the combination of medical therapy, education and medical consultation as effective. The patients’ satisfaction with the therapeutic outcome was on average 6.8 ± 2.0 on an 11-point scale. The question whether patients would recommend MPT to other patients was rated as definitely positive by 72% of the patients.

## Discussion

In the present study, most outcome parameters showed significant or highly significant changes following MPT, with significant reduction of the characteristic pain intensity, improvement of the perceived disability and increase of the subjective well-being, as well as reduction in all three DAS scales. In the evaluation of the MPT as a PROM [34], physiotherapy was judged the most effective therapeutic measure by the patients. Among the medical treatments, counselling consultations were considered most effective.

MPT has been shown as an effective procedure for the treatment of chronic pain syndromes, whereby it was significantly superior to no treatment or conventional pain treatment ^20^. This was shown particularly in studies regarding chronic low back pain. For instance, MPT programs in Bavaria and Saxony achieved positive and stable treatment results. Pain intensity was reduced on average by 1.5 points on the NRS [35, 36]. Buchner et al. reported a mean pain reduction of nearly 1.7 points 6 months after therapy [37]. The results of the present study are somewhat below these observations at 1.4 points. A possible explanation for the smaller pain reduction might be that MPT programs were originally designed for low back pain and up to now place too little focus on facial pain. Therefore, an adjustment of the therapeutic measures particularly to the treatment of facial pain might increase the efficacy of MPT for PIFP.

The study collective showed the typical distribution with female predominance [38] and higher incidence in middle and higher age groups [3]. A high percentage of the patients (96%) had psychiatric comorbidities, which is a frequent finding in patients with PIFP [39] and may indicate the possible association of the two diagnoses. Among the concomitant medications, antidepressants and anticonvulsants were used most frequently, corresponding to current recommendations [7]. Most frequent accompanying measures were consultations of further disciplines (i.e. dental / oral surgical) reflecting intensified interdisciplinary cooperation [3]. Nearly half of the patients reported prior dental interventions, with tooth extractions in first place. Invasive interventions are frequently associated with PIFP and due to frequent lack of improvement of pain, it has been recommended that such dental interventions be avoided in these patients [13].

The effects of MPT on psychological parameters such as anxiety and depression may be considered equally important to the pain-relieving effect*.* In the present study the pre-post comparison regarding the depression, anxiety and stress scales has considerable limitations due to the changes in the versions (HADS vs. DASS) [24] and due to the small sample size. In a study on 198 patients with chronic pain, Schütze et al. observed an improvement in the average depression scale value of 1.3 and the anxiety scale value of 1.5 1 year after MPT [35]. A study on MPT for fibromyalgia showed differences of 0.7 (depression-scale) and 1.6 (anxiety-scale) [40]. In the present study, depression scales were reduced by 2.4 and anxiety scale by 2.9. These results are interesting, as there is a strong link between depression and chronic pain, which have been shown to be interrelated [41]. Anxiety can also have an impact on the behavior of pain patients in the sense of fear-avoidance-beliefs [42].

The results as recorded by the DASS hint at a positive impact of MPT. In patients with temporo-mandibular disorders it has been shown that a multidisciplinary pain treatment approach is required due to psychological distress [43]. The impact of therapy on the habitual well-being (MFHW) was reflected in the increase of all single item scores, as well as in the sum scores after therapy. The MFHW sum score increased from 10.8 prior to MPT to 17.6 after therapy. A sum score of 10 is considered as conspicuous, while persons without impairment on average reach a sum score of 20 [33]. This hints at a positive treatment effect induced by MPT. Nonetheless, the decrease in pain rating as well as the amelioration of the DASS ratings cannot be attributed to MPT with certainty, without detailed knowledge about the treatments patients underwent during the follow-up. A chart survey showed that -with few exceptions- all patients had regular psychotherapy in the course after MPT. In all cases under study, the continuation of psychotherapy had been proposed at time of discharge. Only few patients had psychotherapy prior to MPT. Thus, part of the effects on outcome might also be attributed to continued psychotherapy and the initiation and continuation of psychotherapy can be interpreted as an effect of MPT.

Patients rated physiotherapy to be the most efficacious therapeutic measure. Also among the single ratings, physiotherapy attained the highest scores of the three measures physiotherapy, psychotherapeutic treatment, medical therapy. This reflects the result of a randomized controlled study (RCT) which demonstrated the efficacy of physiotherapeutic exercises for orofacial pain [44]. Apparently, the combination of movement exercises and further treatment measures is also effective in the treatment of PIFP [21]. However, it is important not to lay the focus only on physiotherapy, but to keep the combination of the single therapeutic measures in view. Multidisciplinary pain treatment, as Pfingsten pointed out: „depends on an adequate mixture of treatment. “ [45]. Future studies should definitely focus on the chronological dosing of MPT and its single elements. The high patient satisfaction with the therapeutic outcome hints at a noticeable efficacy of MPT for PIFP.

### Limitations

A number of limitations have to be discussed: first, the small sample size reduces the generalizability of the results. However, as PIFP is a very unusual diagnosis, higher sample sizes are difficult to acquire at a single institution. In fact, many studies of PIFP deal with comparable or smaller sample sizes [46–49]. Further, the outcomes may only be attributed to the specific therapeutic program at our institution although this is similar to corresponding programs in Germany. In addition, due to the retrospective study design, a selection bias cannot be ruled out. Moreover, no control group including healthy subjects or patients undergoing conventional therapy was conducted. A strength of the study is the thorough patient selection according to IHS criteria. Standardization of the classification of facial pain syndromes has been called for on different occasions [9, 10]. Nonetheless in many institutions, including ours, there is some variability in how PIFP is diagnosed and coded in the clinical routine. Therefore in the present study considerable effort was undertaken to ensure that only patients entirely fulfilling the criteria of PIFP were included. With the new edition of the German pain questionnaire, changes were made in single measures, such as HADS-D and DASS. The comparability of these measures, however, has now been proven [24]. Methodologically, the data acquisition at different time points could be criticized. However, as the interviews took place at least 6 months after therapy, there was at least a sufficient time interval after therapy. Moreover objection could still be raised concerning the duration of follow-up possibly leading to recall bias. However, as MPT strives to obtain a lasting change in patients’ behavior, beneficial therapeutic effects often occur only in the course of time after MPT. Therefore the effect of MPT may be assessed more properly after a longer time span.

It could be objected that the details of individualized therapy in MPT (i.e. of dosage of medication or exact physiotherapeutic or psychological technique in use) are not standardized. This probably holds true for most studies on MPT. MPT however should not only be understood as a completely standardized therapy. Rather it could be characterized as an organizational structure allowing pain therapy under consideration of the individual comorbidities [18, 50]. Further, it seems that the interdisciplinary decision-making accounts for much of the therapeutic effect.

A strength of the study is the detailed examination of all patients, comprising the investigation of multiple parameters and the analysis of the effects of MPT on different areas of life.

In summary, the present study shows beneficial outcomes in patients with PIFP following MPT. MPT therefore may also be efficacious in PIFP, corresponding with many other chronic pain syndromes. These findings should be replicated in further studies with numbers of participants. Due to the rarity of the disorder a multicentric controlled study design would be desirable. These studies should also aim to analyze how the individual therapeutic measures, respectively their composition in the context of MPT, can contribute to further increase the efficacy of MPT.
